# Changes in Dietary Patterns and Environmental Footprints Among University Students: A Retrospective Study

**DOI:** 10.3390/ijerph23010083

**Published:** 2026-01-07

**Authors:** Gordana Kenđel Jovanović, Greta Krešić, Elena Dujmić, Mihaela Sabljak, Sandra Pavičić Žeželj

**Affiliations:** 1 Department of Health Ecology, Teaching Institute of Public Health of Primorsko-Goranska County, 51000 Rijeka, Croatia; sandrapz@uniri.hr; 2 Department of Health Ecology, Faculty of Medicine, University of Rijeka, 51000 Rijeka, Croatia; mihaela.sabljak7@gmail.com; 3 Department of Food and Nutrition, Faculty of Tourism and Hospitality Management, University of Rijeka, 51410 Opatija, Croatia; gretak@fthm.hr (G.K.); elena.dujmic@fthm.hr (E.D.)

**Keywords:** dietary patterns, environmental impact, nutrition transition, planetary health diet, sustainable nutrition, university students

## Abstract

**Highlights:**

**Public health relevance—How does this work relate to a public health issue?**

- Students’ shared environments, limited options, and lifestyle constraints lead to unhealthy, unsustainable diets that raise obesity and chronic disease risks.
- Rising dietary environmental footprints contribute to climate-related public health challenges.

**Public health significance—Why is this work of significance to public health?**

- Long-term data show a nutrition transition of declining adherence to health-promoting, sustainable dietary patterns among university students.
- A syndemic relationship between unhealthy diets and environmental decline underscores urgent integrated public health and sustainability approaches.

**Public health implications—What are the key implications or messages for practitioners, policy makers and/or researchers in public health?**

- Universities should improve food environments, affordability, and accessibility to support healthy, sustainable choices of students.
- Aligning nutrition and sustainability goals and promoting nutrition literacy to make effective behavioral change, guiding future research and institutional planning are needed.

**Abstract:**

Background: University students are often exposed to environments that encourage unhealthy eating, but universities can promote better health and sustainability by making sustainable food options more accessible. Methods: Temporal changes in dietary patterns and environmental footprints of 1684 students at the University of Rijeka, Croatia, over a 16-year period (2009–2025) were retrospectively analyzed using data from 3 cross-sectional studies. Results: A significant transition toward less sustainable diets, increased consumption of animal-based foods, and proinflammatory eating habits was observed (both *p* < 0.001). Adherence to the Mediterranean and Planetary Health Diet declined over time (*p* < 0.001), followed by increased prevalence of overweight and obesity. Three dietary patterns were identified: high fruit and vegetable intake, consistently high milk and dairy consumption, and lower-to-moderate intake of all other food groups with temporal variation. Consumption of most food groups increased, leading to higher water and ecological footprints. Only the intake of fruits, vegetables, whole grains, and fish declined, which corresponded with reduced carbon footprints for these and a few other food groups, while the environmental impact of other foods significantly increased (all *p* < 0.001). Gender, diet quality, and a proinflammatory diet were significant predictors of dietary environmental footprints. Conclusions: The findings underscore the need for systemic changes and addressing barriers at the university level to support sustainable eating behaviors. This study offers valuable insights for policymakers, educators, and researchers, which aim to help students become health-conscious and environmentally responsible consumers. Further research is needed to explore the broader factors influencing dietary choices and the long-term impact of future institutional interventions.

## 1. Introduction

Diet-related health and disease outcomes are influenced by food systems, evolving dietary patterns, and their environmental impacts [1]. These patterns result from a complex interplay of economic, demographic, environmental, and cultural factors, affecting both food choices and broader lifestyles [2]. Dietary behavior is shaped by individual factors (preferences, knowledge, and skills), the micro-environment (family, workplace, school), and the macro-environment (social norms, food culture, economics, and policies) [3]. The nutrition transition, marked by the global shift from plant-based diets to ultra-processed, energy-dense foods, is driven by globalization, urbanization, incomes, and advances in food technology and marketing, contributing to the rise in noncommunicable diseases and obesity. However, strong policy and social action can mitigate these trends [4].

The EAT-Lancet Commission’s planetary-health diet addresses the dual challenges of noncommunicable diseases and environmental sustainability by promoting plant-based diets and reducing environmental impacts [5]. As humanity has surpassed six of nine planetary boundaries, particularly climate change, land-system change, and biodiversity loss, these issues now pose urgent threats, demanding systemic action [6]. Climate change and noncommunicable diseases are converging in a syndemic, requiring integrated action across health, environmental, and policy domains [7]. The urgent need for accepting the sustainable, health-conscious dietary patterns is underscored by the alarming trend of exceeding planetary boundaries [6], highlighting the important connection between public health and environmental preservation. Transitioning to sustainable dietary patterns is a great challenge [8,9,10]. Analyzing nutrition transition, environmental footprints, and stages of the epidemiologic transition offer valuable insights into how societies adapt to evolving health conditions, with shifting health and disease patterns responding to global changes [11]. Ensuring a sustainable nutritional transition within planetary boundaries is timely and crucial for promoting intergenerational health development and addressing climate and eco-environmental health risks, through strategies that minimize transgressions and integrate human health with environmental sustainability [12]. Sustainable healthy diets are those which promote health and wellbeing, minimize environmental impact, ensure accessibility and cultural acceptability, and aim to support optimal growth, prevent malnutrition and diet-related diseases, and preserve biodiversity and planetary health by integrating all sustainability dimensions [8]. Currently, in most EU countries, where the Farm to Fork strategy aims to transition to sustainable diets, current consumption patterns still exceed recommended intakes of calorie-dense foods and are low in fruits, vegetables, whole grains, legumes, and nuts [13].

University students are at an important life stage when long-term dietary habits are formed, yet they often adopt unhealthy eating behaviors due to specific shared environments, limited food options, and lifestyle constraints [14,15,16]. While they have a significant role in shaping the sustainability of their food systems, their awareness level, financial constraints, and restricted access to food options can hinder their adoption of sustainable food choices. Addressing these barriers, such as raising awareness and making sustainable food options more accessible and affordable, is essential to promoting sustainable dietary practices within this population. The intersection of environmental and dietary health among youth is now an important key area for intervention and future research [14,15,16], examining dietary patterns, environmental impacts, educational methods, behavioral barriers, and institutional interventions [17]. Universities are important in shaping future consumer behavior, making dietary sustainability a priority, with holistic approaches, such as combining sustainable food practices, nutrition education, and systemic changes, which are essential for fostering healthier eating habits [17]. Studies among Croatian university students consistently reported low dietary quality, with low intake of fruits, vegetables, and fish, frequent breakfast skipping, and limited nutrition knowledge, indicating prevalent unhealthy eating behaviors [18,19,20,21,22,23,24]. A strong positive correlation between nutrition knowledge and healthy dietary intake was previously observed [20]; however, recent findings suggest a weakening of this association. Despite students’ moderate awareness of the Mediterranean diet benefits, adherence no longer aligns with knowledge levels [24]. This growing gap between awareness and habits highlights how factors such as food accessibility, affordability, taste preferences, and lifestyle pressures often outweigh knowledge in shaping behavior [15]. Abreu et al. likewise found high nutrition literacy of university students but low Mediterranean diet adherence, emphasizing that knowledge alone rarely motivates behavioral change [25]. Similar trends are observed internationally. Increased nutrition literacy does not consistently change into adherence to sustainable diets such as the Mediterranean or Planetary Health Diets. A broader nutrition transition in Europe has become more evident, as evidenced by growing adoption of Westernized, convenience-based eating patterns influenced by globalization and modern lifestyles [26], where significant improvements in diet quality and sustainability are essential to achieve the desired health and environmental goals across Europe. Among Croatian students, the weakened nutrition knowledge–diet relationship [24] probably reflects environmental and social influences, such as wide use of fast food, time constraints, psychological stress, and limited access to healthy, affordable campus options. Behavioral determinants such as affordability, convenience, self-regulation, ethics, and social norms remain key modifiable factors [17]. Food environments, time pressures, and social influences that students are exposed, moderate how nutritional knowledge changes into dietary habits, underscoring the need for interventions that go beyond education to foster more sustainable behavior.

According to the above, this study aims to retrospectively analyze changes in dietary patterns and the nutritional sustainability of students of the University of Rijeka, Croatia, to enhance understanding of observed nutrition transition, and to assess the relationships between dietary choices, health-related aspects, and environmental outcomes. Furthermore, it aims to explore the association between dietary sustainability and students’ socio-demographic characteristics, based on available data. The findings could provide valuable insights into long-term changes in university students’ dietary behaviors and their implications for both human health and environmental sustainability. This research may serve as an evidence base for developing university-level and public health strategies to adopt sustainable and healthy dietary habits among young adults.

## 2. Materials and Methods

### 2.1. Study Design

Based on the predefined inclusion criteria, a targeted literature search identified three cross-sectional quantitative studies [20,21,23] published between 2009 and 2025 that examined dietary habits among students at the University of Rijeka. These studies represented the eligible research, as intervention-based, clinical, and non-student studies were excluded. Accordingly, the present study used a retrospective, integrative analysis of these cross-sectional studies [20,21,23], which investigated dietary habits and nutrition-related aspects among students of the University of Rijeka, Croatia, which is located on the northern Adriatic coast of Croatia, a region that is traditionally characterized by a Mediterranean dietary heritage. The selected studies collectively span a sixteen-year period (2009–2025), providing insight into temporal changes in dietary patterns and nutritional sustainability within this population. The retrospective design allows for comparative synthesis of existing quantitative findings to identify evolving trends, determinants, and sustainability dimensions of student dietary behaviors. This study was conducted in accordance with the ethical standards of the University of Rijeka (Code of Ethics; CLASS: 011-01/18-01/17), the Declaration of Helsinki, and the European Code of Conduct for Research Integrity issued by ALLEA—All European [27]. All included studies had obtained prior ethical approval, which permitted the use of their data for secondary analyses. As the present study relied exclusively on previously published and anonymized data, no additional ethical approval was required. In each of the original studies, participants were fully informed about the study objectives and procedures and provided written informed consent prior to participation. All study protocols were approved by the relevant ethical authorities and adhered to established ethical principles.

### 2.2. Data Analyses

Quantitative and qualitative data were extracted from each database [20,21,23], including students’ characteristics (gender, age, level of study, lifestyle habits, anthropometric variables) and dietary variables. Survey methods of each study are described elsewhere [20,21,23]. Since the authors of this retrospective study were also the authors of selected studies [20,21,23], it was not necessary to obtain author approval for data analyses. A descriptive and comparative analysis was conducted to identify nutrition transition trends across time, dietary quality, and environmental footprints, and to evaluate changes in adherence to healthy and sustainable dietary models, along with the associations with socio-demographic characteristics.

### 2.3. Dietary Assessment

All studies used a semi-quantitative Food Frequency Questionnaire (FFQ) to assess habitual dietary intake among university students. Krešić et al. [20] used a FFQ comprising 97 food items, in which students reported their usual frequency of consumption and average portion sizes over the preceding twelve months. Pavičić Žeželj et al. [21] applied a 50-item FFQ, asking students to note their habitual consumption frequency and average portion sizes of the listed foods during the previous week. Similarly, Kenđel Jovanović et al. [23] used a 98-item FFQ referring to food frequency intake and average servings over the past week. All studies calculated dietary energy and nutrient intakes using the Croatian food composition database [28]. From the generated databases that collected data from the FFQs of each study [20,21,23], individual food, energy, and nutrient intakes were further analyzed.

### 2.4. Dietary Indices

To evaluate students’ dietary patterns based on their nutritional quality, health, and sustainability potential, and environmental impact, dietary indices that address both health and sustainability, such as the Mediterranean Diet Score, Planetary Health Diet Index, and Dietary Inflammatory Index, were used.

The Mediterranean diet was chosen as an indicator of diet quality due to its well-established health benefits and recognition as a sustainable dietary pattern [29,30]. Adherence was assessed using the nine-component Mediterranean Diet Score (MDS), which assigns points based on higher intake of healthy foods (e.g., vegetables, fruits, legumes, fish, and unsaturated fats) and lower intake of meat and alcohol, yielding a total score from 0 to 9 classified as low (≤3), moderate (4, 5) or high (≥6) adherence.

Adherence to a sustainable diet was evaluated using the Planetary Health Diet Index (PHDI), developed by Cacau et al. [31]. The PHDI comprises 16 components, each scored between 5 and 10 points for a total of 0 to 150, including major food groups and specific vegetable ratios to reflect alignment with a diet that supports both human and planetary health. Based on the total PHDI score of each study database, they are further classified into PHDI quartiles.

The Dietary Inflammatory Index (DII) was used to assess the overall health-related inflammatory potential of students’ diets [32]. DII scores were calculated using 36 [20] and 42 [21,23] dietary components, depending on the available data from the FFQ, and following the standardized Shivappa et al. [33] protocol. In brief, for each dietary parameter, a z-score was calculated by standardizing each student’s daily intake to the global mean and standard deviation, then converted to a centered percentile and weighted by its inflammatory effect score. These values were summed to obtain each student’s overall DII score. The total DII scores were classified as anti-inflammatory (DII ≤ 0) or pro-inflammatory (DII > 0). The Danish [34] and American [35] food composition databases were used for certain nutrients needed for the DII calculation.

### 2.5. Environmental Impact Indicators

To assess the environmental impact of students’ diets [20,21,23], data from two publicly available life cycle assessment (LCA) databases were used. These databases offer standardized and comprehensive environmental impact estimates across a wide range of food items and include multiple environmental indicators [36,37]. Environmental impact values from both sources were integrated and linked to the quantities of individual foods, composite dishes, and beverages reported by each student’s FFQ data to estimate diet-related environmental burdens. The primary indicators evaluated were carbon footprint (kg CO_2_ equivalents), ecological footprint (m^2^ × year), and water footprint (m^3^).

### 2.6. Statistical Analysis

Statistical analyses were made using TIBCO Statistica, v. 13.3.0 (TIBCO Software Inc., Palo Alto, CA, USA; 2017). The Kolmogorov–Smirnov test was used to validate the variables’ normality. Comparisons of categorical variables were conducted using the Chi-square test. Continuous variables were analyzed using the Student’s *t*-test or Mann–Whitney U test for comparisons between two groups, and by analysis of variance (ANOVA) or the Kruskal–Wallis H test for comparisons involving more than two groups, as appropriate. Cluster analysis was performed to group food categories based on similarities in intake values across the study periods (2009–2025), using cluster centers to characterize different dietary consumption patterns. Multiple linear regression was used to assess the association between sociodemographic and lifestyle factors (age, gender, nutrition status, physical activity level, study level, and smoking status) and different types of environmental footprints (carbon, water, and land use footprints). Each type of footprint was analyzed as the dependent variable in a separate model, with all other variables serving as independent predictors. This approach enabled control for potential confounders (i.e., age (≤21 years vs. >22 years), gender (male vs. female), level of study (undergraduate vs. graduate), nutrition status (underweight, normal weight, overweight, obesity), physical activity level (low, moderate, vigorous), smoking status (yes vs. no), PHDI quartiles (1st, 2nd, 3rd, 4th quartile), MDS adherence (low, moderate, high), DII potential (proinflammatory vs. anti-inflammatory), and enabled the evaluation of the single influence of each predictor on the different types of environmental footprints. All statistical tests were two-sided, with a *p*-value of less than 0.05 considered statistically significant.

## 3. Results

### 3.1. Socio-Demographic, Lifestyle, and Dietary Factors

Table 1 shows the demographic, lifestyle, and selected dietary characteristics of 1684 University of Rijeka students across three studies [20,21,23]. Women predominated in earlier studies (both 74% in 2009 and 2018), while significantly less in 2025 (46%, *p* < 0.001). Although on average, students were of similar age and almost equally distributed across age groups (≤21 years: 45% vs. >22 years: 55%), there were significantly more older students in the 2009 and 2025 studies than in the 2018 study (*p* < 0.001). While the total sample and the 2025 study had the same proportion of undergraduate and graduate students, the 2009 study had more graduate students (62%), and the 2018 study had more undergraduate students (75%) (*p* < 0.001). The BMI increased over time (22.08 kg/m^2^ in 2009, 22.43 kg/m^2^ in 2018 and 24.11 kg/m^2^ in 2025, *p* < 0.001), with significant increase in the prevalence of overweight and obesity in the 2025 study (overweight 13% in 2009 to 28% in 2025, obesity 1% in 2009 to 7% in 2025, *p* < 0.001). However, there was an increase in the prevalence of high-intensity physical activity (55% normal and high physical activity in 2009, 80% in 2025, *p* < 0.001), while smoking habits decreased (34% in 2009, 25% in 2025, *p* < 0.001). High adherence to the Mediterranean Diet and Planetary Health Diet significantly declined over time (MDS 49% in 2009, 7% in 2018, and 15% in 2025, *p* < 0.001; PHDI 34% in 2009, 36% in 2018, and 30% in 2025, *p* < 0.001). Accordingly, students’ diet became over time significantly more proinflammatory (DII > 0 were as follows: 46% in 2009, 91% in 2018, and 62% in 2025, *p* < 0.001). Similarly, the diet’s environmental impact regarding water and land use showed a significant upward trend, with highest in the 2025 study (all *p* < 0.001), although the highest carbon footprint had average diets in the 2009 study (*p* < 0.001). Average dietary energy intake was significantly lower in the 2018 study than others (*p* < 0.001). That study noted the highest intake of animal protein, a higher saturated fat ratio, and the lowest calcium, iron, and folate intake than in other studies (all *p* < 0.001), except for vitamin D intake, which was the lowest in the 2025 study. The 2009 study showed the most favorable nutrient intakes (dietary fiber, calcium, iron, more plant-based protein; healthier fat ratio, all *p* < 0.001) than other studies, while the 2025 study showed the highest average intake of cholesterol and folate (all *p* < 0.001) than other studies.

**Figure 1:**
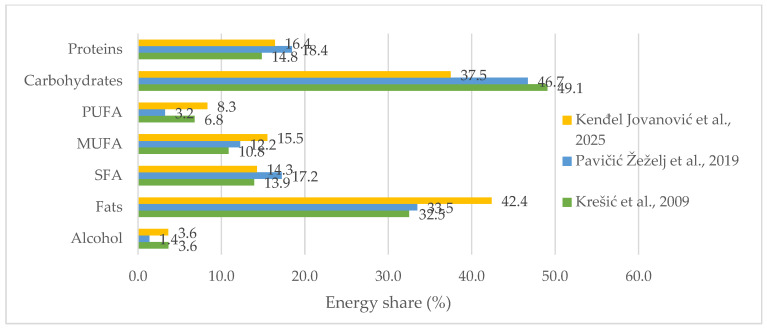
Macronutrient energy share among university students (n = 1684) from Krešić et al., 2009 [20]; Pavičić Žeželj et al., 2019 [21]; Kenđel Jovanović et al., 2025 [23].

The energy share of macronutrients in the 2009 study was more favorable for protein, total fats, and carbohydrates than in later studies (all *p* < 0.001) (Figure 1). From 2009 to 2018, the shares of carbohydrates, PUFA, and alcohol in energy decreased, while the shares of total fat, SFA, MUFA, and protein increased (all *p* < 0.001). In the 2025 study, students’ average diet had a significantly increased energy share of fats, especially of the MUFA and PUFA (all *p* < 0.001), while that of carbohydrates decreased (*p* < 0.001). Protein energy share significantly decreased from 2018 to 2025 (*p* < 0.001), while alcohol energy share returned to those values noted in 2009 (Figure 1).

### 3.2. Food Group Intake and Environmental Impact Changes

Table 2 presents dietary changes in food group intake across the three study periods and associated environmental impact. Over the 2009–2025 period, consumption increased for most of the 14 food groups, likewise associated increase in their environmental footprints. Declining intake was only in fruits, vegetables, whole grains, and fish (all *p* < 0.001), whereas legume and egg intake increased (both *p* < 0.001). Fish (*p* < 0.001) and animal fats (*p* = 0.002) intake remained relatively similar. Tubers and potatoes (*p* < 0.001), and milk and dairy (*p* = 0.002) intake decreased from 2009 to 2018 but increased significantly in 2025, while vegetable oils’ intake increased constantly (*p* < 0.001), and animal fat (*p* = 0.002) intake remained similar. Cluster analysis identified three different dietary intake patterns across the examined studies (Table 2). Cluster 0 comprised food groups with generally low to moderate consumption levels, which included nuts, legumes, whole grains, eggs, fish, meat and meat products, fats, oils, and added sugars. Cluster 1 was characterized by consistently high consumption of fruits and vegetables, representing a plant-based dietary pattern. Cluster 2 consisted exclusively of milk and dairy products, which showed the highest intake values and formed a distinct cluster across all study years (2009–2025). Over sixteen years, carbon footprint significantly decreased for legumes, fruits, vegetables, tubers and potatoes, eggs, red meat, fish, animal fats, and added sugars (all *p* < 0.001), and increased for nuts and peanuts, whole grains, poultry, milk and dairy, and vegetable oils (all *p* < 0.001) (Table 3). Water footprint significantly increased for almost all foods, for nuts and peanuts, fruits, legumes, vegetables, tubers and potatoes, whole grain, eggs, red meat, poultry, milk and dairy, vegetable oils, animal fats, and added sugars (all *p* < 0.001), and remained the same for fish (*p* < 0.001). The ecological footprint significantly decreased for fruits, vegetables, legumes, whole grains, and fish (all *p* < 0.001), increased for nuts, legumes, tubers and potatoes, milk and dairy, vegetable oils, red meat, poultry, and added sugars (all *p* < 0.001), and remained stable for the animal fats (*p* < 0.001) (Table 3).

### 3.3. The Association Between Environmental Sustainability and Students’ Characteristics and Lifestyle

The association between dietary carbon footprint and students’ characteristics is presented in Table 4. Gender was a significant predictor of students’ dietary carbon footprint, where women had a significantly lower footprint than men, with increasing effect size over time (2009 β = −462.65, *p* = 0.007; 2018 β = −987.98, *p* < 0.001; 2025 β = −1778.82, *p* < 0.001) (Table 4). Age, nutritional status, physical activity and smoking habits, and level of study showed no significant associations with dietary carbon footprint (all *p* > 0.05). The PHDI index was significantly and positively associated with carbon footprint in the 2018 study (β = 193.39, *p* = 0.034) but negatively in the 2025 study (β = −568.02, *p* = 0.012), showing diet-specific differences associated with students’ characteristics and their change over time. The MDS had a significant positive impact only in the 2009 study (β = 444.31, *p* = 0.001), with insignificant effects in other studies. A more proinflammatory diet was a significant predictor for higher carbon footprint, with a significant increasing time-related trend (2009 β = 1906.08, *p* < 0.001; 2018 β = 2106.11, *p* < 0.001; 2025 β = 3271.46, *p* < 0.001). Women’s average diet had significantly lower water footprints, with the largest gender difference in 2025 (β = −1302.98, *p* = 0.001) (Table 5). In studies from 2009 and 2025, higher PHDI scores were significantly associated with lower water footprints (β = −132.05; *p* < 0.001; β = −427.29; *p* = 0.011, respectively), whereas the MDS showed a significant positive association only in the 2009 study (β = 148.85; *p* = 0.001). A more proinflammatory diet was associated with higher water footprints, with effect sizes significantly increasing over time (2009 β = 525.94, *p* < 0.001; 2018 β = 1206.90, *p* < 0.001; 2025 β = 2564.66, *p* < 0.001). Age, nutrition status, physical activity habits, and study level showed no significant associations with dietary water footprint (all *p* < 0.001). Land use also showed a time-related trend, with gender and the DII as significant predictors of students’ ecological footprints (Table 6). Women’s average diet had a significantly increasing association over time (2009 β = −0.66, *p* < 0.001; 2018 β = −2.16, *p* < 0.001; 2025 β = −3.39, *p* < 0.001). Nutritional status was a significant predictor only in the 2009 study, where it was negatively associated (β = −0.33; *p* = 0.027), while it showed a positive, but insignificant association over time (2018 β =0.50, *p* = 0.179; 2025 β = 0.08, *p* = 0.885). Higher diet quality assessed with PHDI was significantly associated with lower land use in the 2009 (β = −0.29; *p* < 0.001) and 2025 (β = −0.87; *p* = 0.018) studies. Mediterranean diet adherence was significantly positively associated with land use only in the 2009 study (β = 0.53; *p* < 0.001). A more proinflammatory diet was the strongest predictor for significant and positive association with land use, with effect sizes significantly increasing over time (β ranging from 1.72 in 2009 to 7.36 in 2025; all *p* < 0.001, respectively).

## 4. Discussion

This retrospective study provides a comprehensive analysis of dietary patterns and their environmental and health impacts among students of the University of Rijeka, Croatia, over a sixteen-year period (2009–2025). The results revealed significant trends in both nutritional quality and sustainability, presenting insights into the evolving challenges and opportunities for promoting healthy and sustainable eating behaviors among young consumers. University students are important for making a change; therefore, to promote the health, wellness, and longevity of youth while ensuring the preservation of planetary health, youth-focused public health programs should be developed through collaborative efforts between universities and public health institutions, incorporating knowledge of evolving food systems, ecological public health, and the principles of planetary health diets.

### 4.1. Change in Dietary Patterns/Nutrition Transition

This study revealed a significant change in students’ dietary patterns over time. Their diet’s adherence to the Mediterranean and Planetary Health diet, both of which are associated with improved health outcomes and reduced environmental impacts [29,30,38,39], significantly declined from 2009 to 2025. In 2009, 49% of students’ diet highly adhered to the Mediterranean diet, whereas this proportion declined to 7% in 2018, followed by a slight increase to 15% in 2025. The adherence to the Planetary Health Diet also similarly but mildly declined over time. The observed decline in adherence to sustainable diets had been followed by increased environmental impact indicators, and by increased proinflammatory potential of students’ diets. These findings align with trends observed in other parts of Europe, where Westernized eating patterns and convenience-based food choices are becoming more prevalent [15,16,26,40]. Even diets proven to be healthy, such as Mediterranean, vegetarian, or vegan diets, can have relatively high environmental impacts when they include resource-intensive foods, such as nuts, avocados, or dairy, rely on imported or out-of-season produce, or incorporate highly processed plant-based products. Thus, nutritional quality does not necessarily equate to low environmental footprint, as factors such as production methods, transport, and food waste substantially influence a diet’s ecological impact. Perrone et al. recently discussed that adherence to the Mediterranean diet is declining across Mediterranean populations due to widespread socio-economic, cultural, and behavioral changes, and framing this trend not as a diminution of the diet’s value but as a public health challenge and opportunity for revitalizing and adapting it within current food systems [41]. Changes in students’ diet across sixteen years, observed in this study, revealed significant fluctuations in food group intake and their associated environmental impacts. The identified dietary clusters indicate a stable dietary structure among students over time. The stability of core components such as fruit, vegetable, and dairy intake suggests that many students maintain these foods of a nutrient-rich diet, which is favorable for healthy dietary habits and long-term health. However, dietary habits among university students are strongly influenced by time constraints, academic pressures, and perceived barriers to healthy eating, such as cost and food preparation skills, which can limit adherence to healthy dietary guidelines [14,15,16]. Furthermore, noticed modification in other food groups may reflect adaptive responses to changing lifestyle demands rather than consistent improvements in diet quality. These patterns align with similar research [14,15,16,24] indicating that while some students maintain health-promoting dietary behaviors, substantial proportions continue to display suboptimal intakes and face structural challenges in achieving balanced diets. The stability of dietary clusters observed in this study suggests that university students maintain a relatively consistent dietary structure over time, despite transitional life circumstances. The sustained central intake of fruits and vegetables aligns with evidence indicating that many students retain health-oriented intentions and basic nutritional awareness, even when faced with structural and personal barriers to optimal eating [15], which may explain the observed temporal fluctuations without complete abandonment of these food groups. The consistently high intake of milk and dairy products highlights their role as convenient, affordable, and culturally ingrained staples within student diets. Dairy foods are frequently selected by students due to their accessibility, minimal preparation requirements, and perceived nutritional adequacy, particularly under conditions of limited cooking skills or restricted time [16]. This pattern supports the interpretation that dairy consumption may serve as a compensatory strategy to meet basic nutritional needs during periods of academic stress or irregular meal patterns. Changes observed within the low-to-moderate intake cluster, reflecting gradual dietary modification rather than a fundamental shift in patterns, are consistent with literature describing adaptive rather than transformative dietary behaviors among university students. Student dietary choices are shaped by a complex interaction of convenience, cost, environmental awareness, and social norms, often leading to incremental adjustments rather than comprehensive dietary change [17]. Such modification may include both healthier and less healthy options, suggesting variability in diet quality rather than a uniform improvement. Specifically, while the intake of nuts and peanuts, fruits, vegetables, whole grains, red meat, poultry, and added sugars declined from 2009 to 2018, the consumption of these foods significantly increased in 2025. The consumption of nuts, peanuts, fruits, vegetables, and whole grains has been associated with reduced inflammatory markers and with a lower risk of chronic inflammatory diseases. This specific potential in reducing inflammation biomarkers such as C-reactive protein and interleukin-6 [42] is due to their content of omega-3 fatty acids, polyunsaturated fats, and antioxidants. Students’ nutrition transition over time showed an improved dietary lipid profile, characterized by a higher proportion of unsaturated fatty acids, which is likely attributed to increased consumption of nuts, peanuts, and legumes. Furthermore, legumes, fruits, and vegetables, as good sources of dietary fiber, contribute to the regulation of postprandial blood glucose and insulin sensitivity, and promote gut health by enhancing microbiota diversity that increases production of short-chain fatty acids with anti-inflammatory effects [43]. Conversely, pro-inflammatory foods such as red meat, poultry, and added sugars promote inflammation, with red meat and processed foods contributing to increased inflammation through saturated fats, heme iron, and advanced glycation end products, while excessive poultry and sugar intake are associated with activation of inflammatory pathways like NF-κB signaling [42]. The increase in consumption of pro-inflammatory foods not only more likely contributes to diet-related diseases such as obesity, cardiovascular diseases, and diabetes [42], but also reflects a shift towards diets that are less aligned with sustainability goals [5,8,44]. The observed increase in overweight and obesity prevalence over time within this study further underscores the implications of transitioned dietary trends for students’ long-term health. Recent research [15,16] indicates that university and college students are experiencing increasing rates of overweight and obesity, often driven by poor dietary patterns, physical inactivity, and environmental factors such as limited access to healthy food options. These trends are concerning because habits formed during young adulthood tend to persist into later life and consequently may increase the risk of chronic diseases, such as type 2 diabetes, cardiovascular disease, and metabolic imbalances [45], and even cause an accelerated biological ageing [46]. A recent systematic literature review exploring barriers in sustainable food consumption [47] highlighted factors like economic constraints, limited availability, knowledge gaps, and psychological barriers that hinder consumer adoption of sustainable diets, which is related to the characteristic students’ lifestyle [14,16,17]. Addressing these challenges requires well-planned strategies such as subsidies, education, improved accessibility, and building trust in labelling systems while also tackling systemic barriers in supply chains and production [47]. In addition, multi-level, context-specific interventions that integrate structural, social, and individual determinants, such as affordability, nutrition literacy, and cooking self-efficacy, may effectively promote the adoption of sustainable dietary practices among youth [48].

### 4.2. Environmental Impact of the Diet

The environmental impact of students’ diets analyzed in this study also showed significant temporal changes, characterized by an increase in dietary environmental indicators over time. Overall, the carbon footprint showed a significant decrease across most food groups, with the exception of whole grains, eggs, fish, milk and dairy, vegetable oils, and poultry, which exhibited increasing trends. The water and ecological footprint values were higher in 2025 compared with 2009 across all food groups, with the exception of the water footprint of the added sugars group. An exception was also observed for fruits, vegetables, and whole grains, whose ecological footprint after sixteen years was lower compared with the initial assessment. Animal-based foods are associated with an increase in all ecological footprints, while plant-based foods, especially unprocessed or minimally processed fruits, vegetables, legumes, grains, and nuts, generally with lower carbon emissions, but some plant-based foods, like grains, nuts, and oils, can have increased water use [49]. Daas et al. recently highlighted that when ultra-processed foods are included in plant-based diet indices, their environmental sustainability depends greatly on the specific composition [50]. This study showed that carbon footprints decreased with the intake of nuts, fruits, vegetables, and reduced red meat, and added sugars consumption, but increased with the consumption of whole grains and poultry. Water footprints increased with most foods, including nuts, fruits, whole grains, eggs, and red meat, while ecological footprints decreased with fruits, vegetables, and whole grains, but increased for red meat, poultry, and eggs. This suggests that observed time-related dietary environmental impact transition may depend on differences in the food type and its processing level within the same food group, rather than on differences in consumption quantities. For example, plant-based ultra-processed foods, such as processed grains, generally have lower greenhouse gas emissions and lack essential nutrients than unprocessed or minimally processed animal-based foods, which have much higher impacts due to production systems and LCA values [36,37,51,52]. Although students over time increased red meat and product consumption, they also significantly increased legume intake, which is a positive trend, as legumes are important protein sources in plant-based diets and have much lower carbon footprints than animal-based foods [53]. Although transitioning to plant-based diets may lower environmental impacts, it is also important to recognize that dietary nutritional quality tends to decline with increasing levels of food processing, regardless of the same food origin, which presents a complex challenge [38,54], and seeks more focused education on whole-food intake. Recently, Kalmpourtzidou et al. reviewed that Mediterranean, healthy, and sustainable diets, despite their nutritional and health advantages, express relatively high environmental impacts, particularly greater freshwater and land use, and especially in southern Europe, compared with diets that further reduce meat and animal-based foods [55], which is in line with this study’s results. This study observed significant and consistent gender differences among students’ characteristics associated with the environmental impact of their diet. Women had a diet with lower carbon, water, and land footprints compared to men. This finding is consistent with other research that showed that men had higher dietary environmental impacts than women, when impacts were expressed per total food intake [56,57,58]. However, although reporting impacts per unit of energy may be more appropriate for certain purposes like subgroup comparisons, it does not account for the effects of overconsumption, which is a major contributor to dietary environmental burden [59]. The increasing effect size of this gender difference over time suggests that women tend to have and remain a healthier dietary pattern. Of all dietary indices, DII was consistently associated with environmental impact, where a more proinflammatory diet was significantly associated with greater environmental impacts, indicating that diets detrimental to health also tend to be less sustainable, with this effect strengthening over time. If young people continue to consume proinflammatory and environmentally unsustainable diets, they may face heightened risks of chronic diseases such as obesity, cardiovascular disease, and diabetes. At the same time, these dietary patterns impair environmental degradation, threatening future food security and ecosystem stability. Therefore, adopting nutrient-rich, sustainable diets among students is critical for the preservation of both health and planetary well-being. The connections between dietary practices, nutrition, the environment, and disease provide valuable insights for public health, such as the results of this study, by highlighting the impact of rapidly evolving food systems, nutrition transition, and related health outcomes. The increased reliance on animal-sourced foods, a significant driver of ecosystem degradation and climate change, exacerbates unsustainable dietary trends and further strains ecological sustainability and biodiversity [5,8,12,54]. Abreu et al. showed that despite the clear evidence of students’ growing awareness of the environmental and health benefits of sustainable diets, a significant gap between knowledge and behavior remains [25]. Factors such as food accessibility, affordability, time pressures, and lifestyle constraints significantly shape students’ dietary choices than knowledge alone, and despite relatively high levels of nutrition knowledge, there is a weak relationship between nutrition literacy and actual dietary habits [24,25]. The growing reliance on convenience foods, fast food outlets, and the increasing prevalence of highly processed foods on campuses are major barriers to adopting healthier, more sustainable diets. Furthermore, the challenges of balancing academic, social, and work commitments leave little time for meal preparation, which further exacerbates reliance on unhealthy food choices. Based on these and this study’s findings, it is necessary to design more effective interventions that support the nutritional transition of students toward more sustainable dietary patterns. Interventions aimed at promoting sustainable eating behaviors must, therefore, go beyond education and focus on creating food environments that support healthy and sustainable choices, such as providing affordable and accessible plant-based options in campus cafeterias and offering incentives for sustainable food practices. Nudging strategies, such as optimized choice architecture, strategic menu design, enhanced visibility and labeling of healthy and sustainable meals, and the integration of sustainability-focused informational materials, have been shown as effective campus food service interventions for promoting healthier and more environmentally sustainable food choices among university students [60,61,62,63]. To overcome this, it is necessary to establish a strong collaboration between public health, universities, and the food system.

### 4.3. Implications for Policy and Future Research

The findings from this study highlight the urgent need for systemic changes at the institutional level to support sustainable eating behaviors among university students. As universities play a pivotal role in shaping the dietary habits of young adults, they have a unique opportunity to influence long-term dietary patterns and foster a culture of sustainability [15,16,17,47,48]. While there is growing interest in sustainable eating among students, focus should be on improving the availability and affordability of healthy, sustainable food options, implementing nutrition education programs that address both health and environmental impacts, and promoting practices that reduce the environmental footprint of campus food systems, by expanding affordable, plant-based, and local food options in dining services, and launching awareness campaigns on the environmental impact of food waste and diet choices. By creating an environment where sustainable food options are more accessible and appealing, universities can help students make lasting improvements to both their health and the planet. Further research is needed to explore the multifaceted factors influencing dietary choices among university students, including the role of social norms, peer influence, and the psychological stressors associated with student life. Additionally, longitudinal studies that track dietary behaviors over long periods could provide deeper insights into the long-term impact of university-level interventions on students’ eating habits and their broader environmental and health outcomes.

### 4.4. Study Limitations

This study has several strengths and limitations. A key strength lies in the use of a comprehensive database compiled from three independent studies [20,21,23], which increased the sample size and enhanced the diversity of participants, thereby improving the robustness and ecological validity of the findings. The availability of data collected across different time points also enabled the identification of temporal patterns. Additionally, the use of FFQs across all studies, while similar in design, allowed for the collection of detailed dietary data. However, differences in the different recall periods and specific food items included in each FFQ could have introduced variability in dietary reporting across the studies. Despite these challenges, the FFQs provide valuable insights into dietary patterns over time. This study also employed the LCA method to evaluate the sustainability of dietary patterns, following international guidelines. This approach provides a robust framework for assessing environmental impacts, contributing to this study’s comprehensive analysis of sustainability. However, a limitation of this method is the absence of standardized LCA databases for representative foods in the national marketplace, which could potentially affect the accuracy and comparability of the results. The lack of region-specific databases means that assumptions were made regarding the environmental impacts of certain food items, which may not fully reflect local agricultural practices or food systems [64]. Furthermore, the retrospective design limits this study in that the data were not originally collected to address more specific research questions, resulting in potential gaps or limitations in key factors, such as food accessibility, affordability, time pressures, nutrition knowledge, and lifestyle constraints of students. Methodological differences among the three included studies [20,21,23] may have introduced heterogeneity that affects comparability. Residual confounding and selection bias may also occur, given the limited control over participant characteristics and contextual factors in the original datasets. Finally, although this study provides insight into time-related trends of university students’ diet, external influences occurring during the periods may limit the contemporary relevance of the findings, and causal inferences cannot be established. Nevertheless, the study findings represent valuable data for the Croatian university context and public health. However, to enable efficient transition to sustainable diets among students, further research from other Croatian universities is needed.

## 5. Conclusions

This study observed a significant nutritional transition among University of Rijeka students towards less sustainable dietary patterns. While certain sustainable dietary practices were noted, such as an increased intake of nuts and legumes, the overall sustainability and health characteristic of the diet was negatively impacted by increased animal-based food consumption and proinflammatory eating behaviors over time. These changes have important implications for both students’ health and the environment. Addressing their barriers to sustainable eating and creating supportive food environments in universities is essential in reversing these trends and promoting healthier, more sustainable dietary behaviors. While core health-promoting components of the diet, such as fruit, vegetable, and dairy intake, appear maintained, limitations related to time, financial resources, food access, and lifestyle demands may restrict more substantial improvements in students’ diet quality. This highlights the importance of university-level environmental and policy strategies to support healthier and more sustainable eating habits among students. This study provides valuable suggestions for policymakers, educators, and researchers working to create a generation of students who are not only health-conscious but also environmentally responsible in their food choices.

## Figures and Tables

**Table 1:** The University of Rijeka’s students (n = 1684) characteristics and lifestyle habits.

	All		Krešić et al., 2009 [20]		Pavičić Žeželj et al., 2019 [21]		Kenđel Jovanović et al., 2025 [23]		*p*-Value ^b^
	n	%	n	%	n	%	n	%	
Number	1684	100	1005	60	455	27	224	13	<0.001
Men	504	30	264	26	119	26	121	54	<0.001
Women	1180	70	741	74	336	74	103	46	
Age ^a^	21.95	1.89	21.97	1.74	21.60	1.94	22.67	2.19	<0.001
≤21 years	754	45	383	38	288	63	83	37	<0.001
>22 years	930	55	622	62	167	37	141	63	
Study level									
Undergraduate	843	50	383	38	342	75	118	53	<0.001
Graduate	841	50	622	62	113	25	106	47	
Body mass index (kg/m^2^) ^a^	22.45	3.13	22.08	2.87	22.43	3.33	24.11	3.50	<0.001
Underweight	85	5	49	5	30	7	6	3	<0.001
Normal weight	1316	78	818	81	359	79	139	62	
Overweight	243	14	126	13	53	11	64	28	
Obesity	40	2	12	1	13	3	15	7	
Physical activity level									
Low	730	43	453	45	232	51	45	20	<0.001
Normal	636	38	428	43	109	24	99	44	
High	318	19	124	12	114	25	80	36	
Smoking (yes)	575	34	340	34	179	39	56	25	0.001
Mediterranean Diet Score (MDS) ^a^	4.78	1.40	5.45	0.92	3.70	1.36	4.00	1.47	<0.001
Low adherence	306	18	24	2	204	45	78	35	<0.001
Moderate adherence	820	49	489	49	218	48	113	50	
High adherence	558	33	492	49	33	7	33	15	
Planetary Health Diet Index (PHDI) ^a^	61.98	13.09	67.84	9.94	52.21	11.72	55.54	13.30	<0.001
1st quartile	297	18	160	16	106	23	31	14	<0.001
2nd quartile	483	29	325	32	101	22	57	25	
3rd quartile	330	20	180	18	82	18	68	30	
4th quartile	574	34	340	34	166	36	68	30	
Dietary Inflammatory Index (DII) ^a^	1.11	2.51	0.18	2.10	3.18	2.02	1.07	2.62	<0.001
Proinflammatory diet (DII > 0)	1012	60	460	46	413	91	139	62	<0.001
Anti-inflammatory diet (DII < 0)	672	40	545	54	42	9	85	38	
Carbon footprint (kg CO_2_ eqv.) ^a^	5.93	2.81	6.80	2.43	4.20	2.34	5.52	3.52	<0.001
Water footprint (m^3^) ^a^	2768.49	1525.48	2380.93	768.22	2696.85	1310.68	4652.83	2674.74	<0.001
Ecological footprint (m^2^ × year) ^a^	7.49	4.47	6.47	2.26	7.46	4.35	11.78	6.58	<0.001
Energy intake (MJ/day) ^a^	8.74	3.05	9.16	2.63	7.40	2.93	9.59	4.03	<0.001
Protein ratio (animal/vegetable) ^a^	2.19	1.25	1.76	0.77	2.91	1.41	2.66	1.70	<0.001
SFA:PUFA ratio ^a^	3.06	2.00	2.17	0.68	5.65	2.08	1.80	0.52	<0.001
Cholesterol (mg/day) ^a^	368.39	216.20	326.96	174.79	377.69	222.59	435.38	280.07	<0.001
Dietary fibers (g/day) ^a^	22.18	9.79	25.76	8.85	14.94	6.97	20.82	10.28	<0.001
Calcium (mg/day) ^a^	966.79	414.49	1083.90	371.27	695.58	358.90	992.26	448.54	<0.001
Iron (mg/day) ^a^	18.67	7.94	21.26	7.45	12.78	5.13	19.03	8.39	<0.001
Vitamin D (mcg/day) ^a^	6.61	6.92	6.60	10.53	6.84	4.06	3.66	2.45	<0.001
Folate (mcg/day) ^a^	253.80	153.79	289.32	152.90	150.35	82.81	304.56	170.83	<0.001

^a^ mean ± SD. ^b^ Ordinal data were tested with a Chi-squared test; numerical data with an ANOVA or Kruskal–Wallis’s test, where appropriate. SFA—saturated fatty acids; PUFA—polyunsaturated fatty acids.

**Table 2:** Dietary patterns and related cluster centers of the University of Rijeka’s students (n = 1684) across study periods (2009–2025).

Food (g/Day)	All		Krešić et al., 2009 [20]		Pavičić Žeželj et al., 2019 [21]		Kenđel Jovanović et al., 2025 [23]		*p*-Value ^a^	Cluster
	Mean	SD	Mean	SD	Mean	SD	Mean	SD		
Nuts and peanuts	4.90	12.72	3.25	5.12	2.32	2.81	22.23	27.21	<0.001	0
Legumes	32.35	43.06	18.21	18.92	53.41	59.95	53.02	53.19	<0.001	0
Fruits	276.67	222.32	369.40	224.04	122.75	110.22	173.29	156.38	<0.001	1
Vegetables	228.67	165.33	278.68	174.64	125.14	94.23	214.60	132.54	<0.001	1
Whole grains	27.21	33.20	32.35	37.29	15.04	22.95	28.86	23.71	<0.001	0
Eggs	22.61	27.32	15.42	18.14	29.84	32.34	40.18	37.08	<0.001	0
Fish	47.07	43.02	49.88	40.94	40.75	45.82	47.34	45.05	0.001	0
Tubers and potatoes	107.74	79.97	104.14	64.98	95.52	83.13	148.75	114.59	<0.001	0
Milk and dairy	390.25	224.13	404.55	222.66	358.90	188.87	389.78	283.52	0.002	2
Vegetable oils	20.33	18.80	12.65	6.57	26.83	23.51	41.57	23.31	<0.001	0
Red meat and products	95.69	87.45	95.99	61.40	64.26	79.77	158.19	147.83	<0.001	0
Poultry and substitutes	62.54	53.04	71.03	51.33	31.79	37.29	86.88	60.77	<0.001	0
Animal fats	11.65	5.58	11.43	4.13	11.55	6.10	12.86	8.98	0.002	0
Added sugars	22.92	23.27	22.48	15.87	12.28	11.89	46.48	43.32	<0.001	0
Cluster centers										
0 (low consumption)			39.71		34.87		62.34			
1 (moderate consumption)			324.04		123.95		193.95			
2 (high consumption)			404.55		358.9		389.78			

^a^ ANOVA or Kruskal–Wallis’s test, where appropriate.

**Table 3:** Environmental impact indicators of food intake in University of Rijeka’s students (n = 1684).

	All		Krešić et al., 2009 [20]		Pavičić Žeželj et al., 2019 [21]		Kenđel Jovanović et al., 2025 [23]		*p*-Value *
	Mean	SD	Mean	SD	Mean	SD	Mean	SD	
Nuts and peanuts									
Carbon footprint (g CO_2_ eqv.)	5.84	13.49	3.24	5.10	2.76	3.09	23.77	29.39	<0.001
Water footprint (m^3^)	35.86	90.55	23.36	36.76	22.98	2.76	158.72	194.17	<0.001
Ecological footprint (m^2^ × year)	0.04	0.11	0.02	0.04	3.33	2.98	0.20	0.25	<0.001
Legumes									
Carbon footprint (g CO_2_ eqv.)	12.93	16.08	14.23	18.27	11.06	12.35	10.92	10.96	<0.001
Water footprint (m^3^)	5.84	9.67	1.76	2.07	5.93	7.32	5.78	6.82	<0.001
Ecological footprint (m^2^ × year)	0.02	0.03	0.01	0.02	0.03	0.04	0.03	0.03	<0.001
Fruits									
Carbon footprint (g CO_2_ eqv.)	412.49	451.75	650.94	446.10	53.39	46.66	72.06	63.55	<0.001
Water footprint (m^3^)	61.63	66.98	39.58	35.08	84.71	75.99	113.63	102.80	<0.001
Ecological footprint (m^2^ × year)	0.14	0.12	0.19	0.12	0.06	0.05	0.08	0.07	<0.001
Vegetables									
Carbon footprint (g CO_2_ eqv.)	289.24	238.82	388.23	245.30	113.70	94.32	201.69	163.35	<0.001
Water footprint (m^3^)	49.71	29.86	53.49	27.84	36.77	28.19	59.08	33.56	<0.001
Ecological footprint (m^2^ × year)	0.26	0.24	0.32	0.27	0.15	0.14	0.18	0.11	<0.001
Whole grains									
Carbon footprint (g CO_2_ eqv.)	58.59	59.17	51.66	55.34	65.54	61.53	75.57	65.77	<0.001
Water footprint (m^3^)	51.73	52.17	46.02	50.46	57.27	51.99	66.09	56.18	<0.001
Ecological footprint (m^2^ × year)	0.07	0.08	0.08	0.10	0.06	0.06	0.07	0.06	0.003
Eggs									
Carbon footprint (g CO_2_ eqv.)	83.07	100.50	56.71	66.71	109.42	119.03	147.78	136.37	<0.001
Water footprint (m^3^)	94.28	114.07	64.36	75.71	124.19	135.10	167.73	154.79	<0.001
Ecological footprint (m^2^ × year)	0.14	0.18	0.10	0.12	0.19	0.21	0.26	0.24	<0.001
Fish									
Carbon footprint (g CO_2_ eqv.)	228.49	278.41	346.37	305.38	45.58	51.63	71.19	63.00	<0.001
Water footprint (m^3^)	0.00	0.00	0.00	0.00	0.00	0.00	0.00	0.00	<0.001
Ecological footprint (m^2^ × year)	0.01	0.01	0.01	0.01	0.00	0.00	0.00	0.00	<0.001
Tubers and potatoes									
Carbon footprint (g CO_2_ eqv.)	47.69	37.20	57.93	38.72	25.93	22.56	45.93	34.84	<0.001
Water footprint (m^3^)	57.91	47.73	48.51	31.44	59.96	52.17	95.92	73.07	<0.001
Ecological footprint (m^2^ × year)	0.05	0.04	0.05	0.03	0.04	0.04	0.06	0.05	<0.001
Milk and dairy									
Carbon footprint (g CO_2_ eqv.)	808.52	499.94	844.45	409.66	689.08	382.10	889.92	889.88	<0.001
Water footprint (m^3^)	544.67	347.46	542.28	298.42	498.47	271.11	649.20	587.54	<0.001
Ecological footprint (m^2^ × year)	0.75	0.56	0.74	0.39	0.65	0.39	0.96	1.12	<0.001
Vegetable oils									
Carbon footprint (g CO_2_ eqv.)	15.50	12.51	15.58	9.91	10.13	8.93	26.05	20.17	<0.001
Water footprint (m^3^)	74.33	71.31	90.41	49.38	42.37	1.97	148.29	100.81	<0.001
Ecological footprint (m^2^ × year)	0.13	0.11	0.16	0.07	0.01	0.01	0.25	0.14	<0.001
Red meat and products									
Carbon footprint (g CO_2_ eqv.)	2574.74	1874.21	2736.51	1772.55	2243.22	1912.85	2522.32	2137.84	<0.001
Water footprint (m^3^)	867.16	878.09	637.85	395.24	908.74	858.13	1811.52	1574.89	<0.001
Ecological footprint (m^2^ × year)	3.70	3.10	3.15	1.69	3.63	3.33	6.34	5.37	<0.001
Poultry and substitutes									
Carbon footprint (g CO_2_ eqv.)	360.56	326.43	295.75	205.00	319.93	217.67	733.91	596.85	<0.001
Water footprint (m^3^)	267.05	272.11	157.41	118.11	344.57	234.44	601.49	453.11	<0.001
Ecological footprint (m^2^ × year)	1.02	1.00	0.65	0.47	1.24	0.85	2.23	1.70	<0.001
Animal fats									
Carbon footprint (g CO_2_ eqv.)	2.06	2.74	2.38	3.11	1.53	1.30	1.67	2.86	<0.001
Water footprint (m^3^)	1.45	2.14	1.86	2.43	0.62	0.58	1.30	2.23	<0.001
Ecological footprint (m^2^ × year)	0.01	0.01	0.01	0.01	0.00	0.00	0.01	0.01	<0.001
Added sugars									
Carbon footprint (g CO_2_ eqv.)	251.85	217.66	299.07	237.18	207.80	177.02	129.49	106.93	<0.001
Water footprint (m^3^)	237.95	268.69	141.80	134.01	389.16	338.80	362.18	355.67	<0.001
Ecological footprint (m^2^ × year)	0.59	0.73	0.34	0.24	1.20	1.11	0.51	0.46	<0.001

* ANOVA or Kruskal–Wallis’s test, where appropriate.

**Table 4:** Associations between dietary carbon footprint (kg CO_2_ eqv.) and characteristics of the University of Rijeka’s students (n = 1684).

	Krešić et al., 2009 [20]				Pavičić Žeželj et al., 2019 [21]				Kenđel Jovanović et al., 2025 [23]			
	β	95% CI		*p*-Value	β	95% CI		*p*-Value	β	95% CI		*p*-Value
Age (1 ≤ 21 years; 2 > 22 years)	−37.97	84.78	−160.72	0.544	−13.64	190.91	−218.19	0.896	−99.91	238.73	−438.55	0.564
Gender (1 men; 2 women)	−462.65	−127.49	−797.82	0.007	−987.98	−511.38	−1464.57	<0.001	−1778.82	−940.44	−2617.20	<0.001
Nutrition status (1 underweight; 2 normal weight; 3 overweight; 4 obesity)	−144.37	164.29	−453.02	0.360	253.84	643.12	−135.43	0.202	−223.13	432.07	−878.33	0.505
Physical activity level (1 low; 2 moderate; 3 high)	84.81	280.65	−111.03	0.396	116.66	359.37	−126.04	0.347	−174.58	487.79	−836.95	0.606
Smoking habits (1 no; 2 yes)	50.79	337.65	−236.07	0.729	266.10	673.43	−141.23	0.201	−84.16	831.91	−1000.23	0.857
Study level (1 undergraduate; 2 graduate)	120.15	559.91	−319.62	0.592	−448.82	464.66	−1362.29	0.336	−394.48	1106.39	−1895.35	0.607
PHDI quartiles (1 Q1; 2 Q2; 3 Q3; 4 Q4)	83.27	222.09	−55.56	0.240	193.39	372.11	14.67	0.034	−568.02	−130.05	−1005.99	0.012
MDS adherence (1 low; 2 moderate; 3 high)	444.31	700.23	188.38	0.001	194.39	531.89	−143.12	0.260	270.95	994.99	−453.08	0.464
DII inflammation potential (1 proinflammatory; 2 anti-inflammatory)	1906.08	2208.89	1603.26	<0.001	2106.11	2845.46	1366.76	<0.001	3271.46	4340.60	2202.32	<0.001

**Table 5:** Associations between dietary water footprint (m^3^) and characteristics of the University of Rijeka’s students (n = 1684).

	Krešić et al., 2009 [20]				Pavičić Žeželj et al., 2019 [21]				Kenđel Jovanović et al., 2025 [23]			
	β	95% CI		*p*-Value	β	95% CI		*p*-Value	β	95% CI		*p*-Value
Age (1 ≤ 21 years; 2 > 22 years)	−20.47	−61.07	20.13	0.323	−28.38	86.40	−143.17	0.628	−58.06	194.91	−311.02	0.653
Gender (1 men; 2 women)	−141.97	−252.82	−31.12	0.012	−610.70	−343.26	−878.14	<0.001	−1302.98	−676.71	−1929.25	<0.001
Nutrition status (1 underweight; 2 normal weight; 3 overweight; 4 obesity)	−17.63	−119.71	84.45	0.735	110.01	328.45	−108.43	0.324	−146.26	343.18	−635.70	0.559
Physical activity level (1 low; 2 moderate; 3 high)	6.74	−58.03	71.50	0.839	39.42	175.61	−96.78	0.571	−58.27	436.52	−553.06	0.818
Smoking habits (1 no; 2 yes)	127.80	32.93	222.66	0.008	89.12	317.69	−139.45	0.445	−176.56	507.75	−860.88	0.614
Study level (1 undergraduate; 2 graduate)	−61.05	−206.50	84.40	0.411	−148.96	363.63	−661.56	0.569	−369.00	752.16	−1490.16	0.520
PHDI quartiles (1 Q1; 2 Q2; 3 Q3; 4 Q4)	−132.05	−177.97	−86.13	<0.001	57.53	157.82	−42.76	0.261	−427.29	−100.13	−754.45	0.011
MDS adherence (1 low; 2 moderate; 3 high)	148.85	64.20	233.50	0.001	122.89	312.28	−66.50	0.204	311.26	852.12	−229.61	0.261
DII inflammation potential (1 proinflammatory; 2 anti-inflammatory)	525.94	425.78	626.10	<0.001	1206.90	1621.79	792.02	<0.001	2564.66	3363.31	1766.00	<0.001

**Table 6:** Associations between ecological footprint (m^2^ × year) and characteristics of the University of Rijeka’s students (n = 1684).

	Krešić et al., 2009 [20]				Pavičić Žeželj et al., 2019 [21]				Kenđel Jovanović et al., 2025 [23]			
	β	95% CI		*p*-Value	β	95% CI		*p*-Value	β	95% CI		*p*-Value
Age (1 ≤ 21 years; 2 > 22 years)	−0.04	0.08	−0.16	0.520	−0.07	0.31	−0.46	0.702	−0.18	0.37	−0.74	0.519
Gender (1 men; 2 women)	−0.66	−0.34	−0.98	<0.001	−2.16	−1.27	−3.05	0.000	−3.39	−2.02	−4.76	<0.001
Nutrition status (1 underweight; 2 normal weight; 3 overweight; 4 obesity)	−0.33	−0.04	−0.63	0.027	0.50	1.23	−0.23	0.179	0.08	1.15	−0.99	0.885
Physical activity level (1 low; 2 moderate; 3 high)	0.05	0.24	−0.13	0.580	0.23	0.68	−0.23	0.329	−0.31	0.77	−1.39	0.576
Smoking habits (1 no; 2 yes)	0.10	0.37	−0.18	0.489	0.46	1.22	−0.30	0.237	−1.82	−0.33	−3.32	0.018
Study level (1 undergraduate; 2 graduate)	−0.05	0.37	−0.47	0.822	−0.84	0.87	−2.54	0.336	−0.74	1.71	−3.19	0.554
PHDI quartiles (1 Q1; 2 Q2; 3 Q3; 4 Q4)	−0.29	−0.16	−0.42	<0.001	0.15	0.49	−0.18	0.368	−0.87	−0.15	−1.58	0.018
MDS adherence (1 low; 2 moderate; 3 high)	0.53	0.77	0.28	<0.001	0.38	1.01	−0.25	0.243	1.05	2.24	−0.13	0.082
DII inflammation potential (1 proinflammatory; 2 anti-inflammatory)	1.72	2.01	1.43	<0.001	3.51	4.89	2.13	<0.001	7.36	9.10	5.61	<0.001

## Data Availability

The data presented in this study are available on request from the corresponding author.

## Abbreviations

The following abbreviations are used in this manuscript:

FFQ	Food Frequency Questionnaire
BMI	Body mass index
PHDI	Planetary Health Diet Index
MDS	Mediterranean Diet Score
DII	Dietary Inflammatory Index
LCA	Life Cycle Assessment
